# Raucherassoziierte interstitielle Lungenerkrankungen: Radiologisches Erscheinungsbild, histopathologische Korrelation und klinische Aspekte

**DOI:** 10.1007/s00117-024-01333-w

**Published:** 2024-07-11

**Authors:** Lisa Jungblut

**Affiliations:** Institut für Diagnostische und Interventionelle Radiologie, Universitätsspital Zürich, Rämistrasse 100, 8091 Zürich, Schweiz

**Keywords:** Rauchen, Bronchiolitis, Interstitielle Pneumonie, desquamativ, Langerhans-Zell-Histiozytose, Interstitielle pulmonale Fibrose, Smoking, Bronchiolitis, Interstitial pneumonitis, desquamative, Histiocytosis, Langerhans-cell, Pulmonary interstitial fibrosis

## Abstract

**Klinisches/Methodisches Problem:**

Die Identifizierung von raucherassoziierten Lungenerkrankungen (SRILD) bei Rauchern ist herausfordernd, da die klinischen Manifestationen unspezifisch sein können und es eine Vielzahl von SRILD gibt, welche nicht nur ineinander übergehen, sondern auch überlappend vorkommen können.

**Radiologische Standardverfahren:**

Bei der Diagnose von SRILD ermöglichen bildgebende Verfahren wie die hochauflösende CT (HRCT) eine Identifizierung charakteristischer Merkmale und dienen daher als wichtiges Puzzlestück zur endgültigen Diagnosestellung.

**Leistungsfähigkeit:**

In Studien zeigte die hochauflösende Computertomographie (HRCT) eine Sensitivität von etwa 80–90 % bei der Identifizierung von raucherassoziierten interstitiellen Lungenerkrankungen (SRILD), während die Spezifität bei etwa 70–80 % liegt. Eine endgültige Diagnose kann häufig nur mithilfe von histopathologischer als auch klinischer Korrelation erfolgen.

**Empfehlungen für die Praxis:**

Regelmäßige Überwachung von Rauchern, besonders bei Atemnot und Husten, sowie eine interdisziplinäre Zusammenarbeit sind bei SRILD entscheidend für die richtige Diagnosestellung und entsprechende Therapie.

## Abstract (en)

**Clinical/methodological issue:**

Identifying smoking-related interstitial lung diseases (SRILD) in smokers is challenging, as clinical manifestations can be nonspecific, and there is a variety of SRILD entities that not only interconnect but can also overlap.

**Radiological standard procedures:**

In diagnosing SRILD, imaging techniques such as high-resolution computed tomography (HRCT) allow the identification of characteristic features, serving as crucial pieces of the puzzle for definitive differentiation.

**Performance:**

Studies have demonstrated that HRCT exhibits a sensitivity of approximately 80–90% in identifying SRILD, with a specificity around 70–80%. The conclusive diagnosis often requires a correlation between histopathological findings and clinical observations.

**Practical recommendations:**

Regular monitoring of smokers, especially when experiencing symptoms like shortness of breath and cough, coupled with a comprehensive diagnosis of SRILD, is crucial for accurate identification and individualized therapy.

Rauchassoziierte interstitielle Lungenerkrankungen (SR-ILD) entstehen durch langfristiges Tabakrauchen. Sie repräsentieren ein Spektrum von interstitiellen Veränderungen, welche sowohl ineinander übergehen als auch gleichzeitig auftreten können. Bei Überlappung von Bildgebungsmerkmalen führt häufig erst die Korrelation von klinischen, radiologischen und histologischen Merkmalen zur richtigen Diagnose. Daher behandelt dieser Artikel verschiedene Aspekte der radiologischen, klinischen und histopathologischen Präsentation der am häufigsten auftretenden SR-ILD.

## Hintergrund

Lungenerkrankungen, die mit dem Rauchen assoziiert sind, zeigen häufig ein vielseitiges Spektrum interstitieller Veränderungen. Während bei der respiratorischen Bronchiolitis (RB-ILD) und der desquamativen interstitiellen Pneumonie (DIP) die Entzündung im Vordergrund steht, führen bei der Langerhans-Zell-Histiozytose destruktive Prozesse im Verlauf zu fibrotischen Veränderungen [15]. Die kombinierte pulmonale Fibrose mit Emphysem (CPFE) sowie die interstitielle pulmonale Fibrose (IPF) repräsentieren oft fortgeschrittene und irreversible Stadien dieses Lungenerkrankungsspektrums. Obwohl jede dieser Pathologien eigenständig auftreten kann, kommt es häufig zu gleichzeitigem Auftreten und fließenden Übergängen zwischen den verschiedenen Krankheitsbildern.

**Abb. 1:**
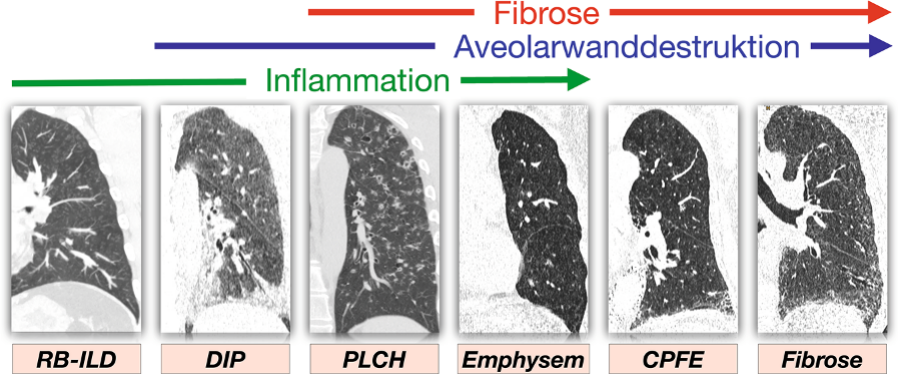
Spektrum von raucherassoziierten interstitiellen Lungenerkrankungen. *RB-ILD* respiratorische Bronchiolitis mit interstitieller Lungenerkrankung, *DIP* desquamative interstitielle Pneumonie, *PLCH* pulmonale Langerhans-Zell-Histiozytose, *CPFE* kombinierte pulmonale Fibrose mit Emphysem, *IPF* idiopathische pulmonale Fibrose

Die bildgebende Diagnostik, insbesondere die hochauflösende Computertomographie (HRCT), spielt eine entscheidende Rolle bei der Erkennung und Charakterisierung dieser interstitiellen Lungenerkrankungen [15, 21]. Die Bandbreite der unterschiedlichen Erkrankungen und der damit verbundene pathologische Prozess werden in Abb. 1 veranschaulicht. Die therapeutischen Ansätze variieren je nach dem Stadium und der spezifischen Ätiologie der Lungenerkrankung. Gemeinsame Maßnahmen können die Raucherentwöhnung, entzündungshemmende Medikamente und in fortgeschrittenen Stadien eine Lungentransplantation umfassen [21]. Eine multidisziplinäre Zusammenarbeit zwischen Pneumologen, Radiologen und Pathologen ist entscheidend für eine präzise Diagnose und entsprechende Behandlungsstrategien.

## Respiratorische Bronchiolitis-assoziierte interstitielle Lungenerkrankung (RB-ILD)

### Epidemiologie und klinische Präsentation

Respiratorische Bronchiolitis ist eine histopathologische Veränderung, die von einer Anhäufung von pigmentierten Makrophagen in den distalen Atemwegen gekennzeichnet ist und praktisch bei allen Rauchern nachweisbar ist. Sie verläuft in der Regel asymptomatisch und ist klinisch von geringer Bedeutung [24]. Wesentlich seltener entwickelt sich bei intensiven Rauchern die respiratorische Bronchiolitis mit interstitieller Lungenerkrankung (RB-ILD). Dabei handelt es sich um eine klinisch-pathologische Entität, die durch pulmonale Symptome, abnorme Ergebnisse von Lungenfunktionstests und bildgebende Auffälligkeiten gekennzeichnet ist. RB-ILD betrifft in der Regel aktive Raucher im Alter von 30–40 Jahren mit einer Raucheranamnese von ca. 30 pack years (py) oder mehr [27]. Es besteht eine leichte Überzahl von Männern. Die häufigsten Symptome sind leichter Husten, Atemnot sowie inspiratorisches Knistern. Die Ergebnisse der Lungenfunktionstests können normal sein oder ein gemischtes obstruktiv-restriktives Muster mit reduzierter Diffusionskapazität zeigen [22].

### Histopathologische Präsentation und radiologische Korrelation

**Abb. 2:**
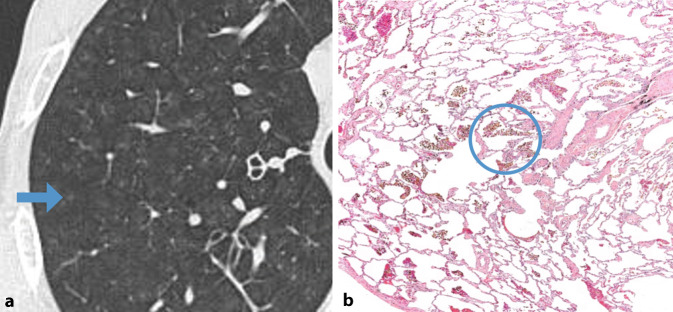
Respiratorische Bronchitis mit interstitieller Lungenerkrankung. Zentrilobuläre Noduli (**a**, *Pfeil*) mit entsprechend pigmentierten Makrophagen in den respiratorischen Bronchiolen (*Kreis*) im histopathologischen Schnitt (**b**)

Eine charakteristische Veränderung in der HRCT bei RB-ILD sind zentrilobuläre Noduli, insbesondere in den apikalen Lungenabschnitten. Zudem können Ground-glass-Opazitäten und eine Verdickung der Bronchialwände vorkommen. Das radiologische Bild der RB-ILD entsteht durch die Ansammlung von pigmentierten Makrophagen in den respiratorischen Bronchiolen und Alveolen [25]. Zudem resultiert die langfristige Exposition gegenüber schädlichen Substanzen in leichtgradigen entzündlichen Veränderungen im interstitiellen Gewebe, die sich durch eine Verdickung der alveolären Septen in den peribronchialen Regionen präsentieren. Ein geringer Anteil der Betroffenen zeigt ein retikuläres Muster, das auf Fibrose hindeutet, jedoch ohne das Vorhandensein von Honigwabenmuster und Traktionsbronchiektasen [14]. Bei der Differenzialdiagnose sind insbesondere die Hypersensitivitätspneumonitis, die desquamative interstitielle Pneumonie (DIP) und eine nichtspezifische interstitielle Pneumonitis (NSIP) zu berücksichtigen. In Abb. 2 sind die radiologischen/histopathologischen Veränderungen ersichtlich.

### Therapie und Prognose

Die Prognose für Patienten mit RB-ILD ist in der Regel günstig. Der Zustand der meisten Patienten bleibt stabil oder verbessert sich. Eine fortschreitende fibrotische Lungenerkrankung tritt nicht auf. Das wichtigste Mittel zur Behandlung von RB-ILD ist der Rauchstopp, jedoch zeigt in Studien eine Minderheit der Patienten trotz Rauchverzicht eine Progression [30]. In den meisten Fällen spielen Kortikosteroide nur eine geringe Rolle, obwohl in Einzelfällen positive Ergebnisse berichtet wurden [27].

## Desquamative interstitielle Pneumonie

### Epidemiologie und klinische Präsentation

Die desquamative interstitielle Pneumonie (DIP) wurde 1965 von Liebow et al. [19] beschrieben. Der Begriff basierte ursprünglich auf der Annahme, dass das dominierende histologische Merkmal die Abschuppung von Epithelzellen sei, wurde jedoch später als Ansammlung von intraalveolären Makrophagen erkannt. Die Inzidenz von DIP bei Rauchern liegt zwischen 58 und 91 %. Die Erkrankung kann jedoch auch bei Nichtrauchern auftreten und wurde mit Bindegewebserkrankungen, rheumatoider Arthritis, Medikamenten wie Sirolimus, Infektionen und dem Konsum großer Mengen von Marihuana in Verbindung gebracht [12]. Die Erkrankung tritt häufiger bei Männern auf, wobei das Geschlechtsverhältnis männlich zu weiblich etwa 2:1 beträgt. Das Erkrankungsalter liegt zwischen 30 und 60 Jahren. Klinisch zeigt sich DIP durch unspezifische Symptome, darunter langsam fortschreitende Atemnot, Husten und Belastungsdyspnoe. Pulmonale Funktionstests zeigen oft eine restriktive Ventilationsstörung, die durch eine Abnahme der Vitalkapazität und des Lungenvolumens gekennzeichnet ist.

### Histopathologische Präsentation und radiologische Korrelation

**Abb. 3:**
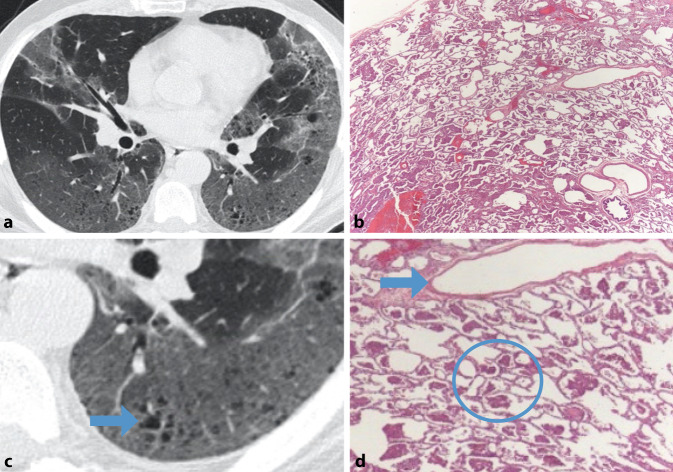
Desquamative interstitielle Pneumonie. Flächige Ground-glass-Opazitäten im Unterlappen beidseits (**a**) mit makrophagengefüllten Alveolen als histopathologischem Korrelat (**b**, **d**, *Kreis*) sowie bereits dünnwandigen Zysten bei fortgeschrittener Destruktion (**c**, *Pfeil*)

In der HRCT zeigen sich diffuse Ground-glass-Opazitäten, vorwiegend in den Unterlappen. Histopathologisch entsprechen diese Veränderungen der Ansammlung von pigmentierten Makrophagen sowie den Produkten makrophagenreicher Desquamation innerhalb der Alveolen. Im Gegensatz zur RB-ILD, welche ebenfalls mit pigmentierten Makrophagen einhergeht, ist die Präsentation in der DIP diffuser und exzessiver [14]. Zudem kann eine leichte Verdickung der interlobulären Septen sichtbar sein, die mit einer milden interstitiellen entzündlichen Reaktion in Verbindung steht. Etwa ein Drittel der Patienten zeigt kleine, klar definierte Zysten (< 2 cm) innerhalb der Ground-glass-Opazitäten. Ein Honigwabenmuster ist ungewöhnlich, wurde jedoch im Rahmen von schwerwiegenden Verläufen beschrieben [18]. Die differenzialdiagnostische Abklärung umfasst RB-ILD, exogen allergische Alveolitis, NSIP und atypische Infektionen [13]. In Abb. 3 sind die radiologischen/histopathologischen Veränderungen dargestellt.

### Therapie und Prognose

Der Goldstandard der Therapie ist die sofortige Raucherentwöhnung, um das Fortschreiten der Erkrankung zu verlangsamen und Symptome zu verbessern. Bronchodilatatoren können zur Atemnotlinderung eingesetzt werden, und in manchen Fällen kann eine Sauerstofftherapie erwogen werden. Obwohl keine randomisierten Studien die Wirksamkeit von Kortikosteroiden nachgewiesen haben, werden sie für Patienten mit fortschreitender Krankheit empfohlen. Bei etwa 25 % der Patienten schreitet die Erkrankung trotz Behandlung fort [27].

## Pulmonale Langerhans-Zell-Histiozytose

### Epidemiologie und klinische Präsentation

Bei der Langerhans-Zell-Histiozytose (PLCH) handelt es sich um eine Gruppe von oft im Kindesalter diagnostizierten Krankheiten meist unklarer Ursache. Hierbei sind Ansammlungen von Langerhans-Zellen in verschiedenen Organsystemen wie Knochen, Lunge, Hypophyse, Schleimhäuten, Haut, Lymphknoten und Leber beteiligt. PLCH betrifft bei Erwachsenen hauptsächlich die Lunge, während außerhalb der Lunge Manifestationen in 5–15 % der Fälle auftreten können [31]. Die Erkrankung ist stark mit Rauchen verbunden (90–100 % sind aktuelle oder ehemalige Raucher), tritt im Alter von 20–40 Jahren auf und präsentiert sich oft mit Husten und Atemnot. Bis zu 25 % der Patienten sind asymptomatisch, während Gewichtsverlust, Fieber, Nachtschweiß und ein spontaner Pneumothorax zu den häufigsten Symptomen gehören. Die pulmonale Funktionstestung (PFT) geht bei 60–90 % der Patienten mit einer Verringerung der Diffusionskapazität einher [31].

### Histopathologische Präsentation und radiologische Korrelation

**Abb. 4:**
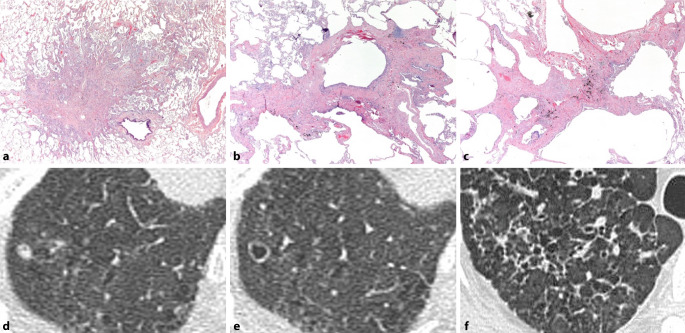
Pulmonale Langerhans-Zell-Histiozytose im zeitlichen Verlauf mit granulomatöser Entzündung (**a, d**, Zystenbildung (**b, e**) und sternförmigen Narben mit Fibrose (**c, f**)

Histopathologisch zeichnet sich PLCH durch eine Ansammlung von Langerhans-Zellen innerhalb der Bronchialwände aus. Diese Ansammlungen bilden Granulome, welche zu einer schrittweisen Zerstörung der Bronchialwände und zur Bildung zystischer Veränderungen führen können. Die histopathologische Untersuchung ist von wesentlicher Bedeutung, um die Diagnose von PLCH zu verifizieren [1]. Radiologisch lassen sich die Befunde direkt mit den histopathologischen Veränderungen korrelieren. Die sichtbaren, primär in den apikalen Lungenabschnitten vorhandenen Noduli entsprechen den Granulomen [29]. Die Ansammlung von Granulomen und der einhergehenden Entzündungsreaktion führt zur Bildung von fibrotischen Knötchen, welche im Verlauf peribronchioläre sternförmige Narben bilden [6]. Dieser Prozess geht mit der Destruktion der Bronchialwände einher und führt zur irregulären Dilatation der distalen Bronchialwege. Längerfristig führt dies zu Kavitationen innerhalb der Noduli. Schließlich bilden sich dickwandige Zysten, welche durch vollständige Destruktion der Bronchialwände sowie durch Verschmelzung zu dünnwandigen Zysten und schließlich zu Bullae fortschreiten [5]. Bei Patienten, die bei der HRCT nur Noduli aufweisen, umfasst die Differenzialdiagnose ein weites Spektrum; Sarkoidose, Silikose, metastatische Erkrankungen und Tuberkulose. Die Verteilung in den oberen und mittleren Lungen sowie die zentrilobuläre Natur der Knötchen bei PLCH sind hilfreiche differenzierende Merkmale. Die zystische Erkrankung bei PLCH sollte von Lymphangioleiomyomatose, Emphysem und IPF unterschieden werden. Im geeigneten klinischen Kontext sind die Befunde der HRCT hochspezifisch und machen weitere Tests überflüssig [4]. In Abb. 4 werden die radiologischen/histopathologischen Veränderungen im zeitlichen Verlauf dargestellt.

### Therapie und Prognose

Das Beenden des Rauchens ist entscheidend und führt bei den meisten Patienten zur Stabilisierung der Symptome. Bei einem erheblichen Anteil der Patienten kann dies die einzige erforderliche Maßnahme sein. Kortikosteroide sind die Hauptstütze der medikamentösen Therapie bei PLCH. Chemotherapeutische Wirkstoffe wie Vinblastin, Methotrexat, Cyclophosphamid, Etoposid und Cladribin werden bei Patienten mit fortschreitender Erkrankung eingesetzt, die nicht auf Kortikosteroide ansprechen oder bei welchen mehrere Organe betroffen sind [29, 31]. Eine Lungentransplantation wird für Patienten mit fortgeschrittener PLCH in Betracht gezogen, die mit schwerwiegender Ateminsuffizienz und begrenzter Lebenserwartung einhergeht. Der natürliche Verlauf ist bei einem einzelnen Patienten variabel und schwer vorhersehbar. Etwa 50 % der Patienten erleben eine günstige Entwicklung mit teilweisem oder vollständigem Verschwinden radiologischer Auffälligkeiten und Auflösung der Symptome. Bei 30–40 % der Patienten persistieren Symptome unterschiedlicher Schwere, und bei 10–20 % tritt ein rezidivierender Pneumothorax oder fortschreitende Ateminsuffizienz mit Cor pulmonale auf. Einige Fälle von Rückfall trotz Rauchstopp wurden berichtet [10].

## Kombinierte pulmonale Fibrose mit Emphysem

### Epidemiologie und klinische Präsentation

Die kombinierte pulmonale Fibrose mit Emphysem (CPFE) ist eine fortschreitende Lungenpathologie mit starker männlicher Prävalenz, begrenzten Behandlungsoptionen und schlechter Prognose. Die komplexe Pathologie umfasst emphysematöse Zerstörung des Lungenparenchyms, interstitielle Fibrose, Veränderungen in der Immunzellzusammensetzung und Umstrukturierung der Blutgefäße. Die Koexistenz von Emphysem und Fibrose wurde erstmals von Cottin et al. 2005 als CPFE benannt [8]. Die Ätiologie von CPFE ist nicht vollständig verstanden, jedoch wird angenommen, dass komplexe Wechselwirkungen zwischen Zigarettenrauch, anderen chronischen Umwelteinflüssen und genetischen Faktoren eine Rolle spielen [9]. CPFE betrifft typischerweise Männer im Alter von 60–80 Jahren mit Rauchgeschichte. Es zeigt sich durch Atemnot bei Belastung, chronischen Husten und Sauerstoffdesaturation. Auffällige Anzeichen sind basales inspiratorisches Knistern sowie apikal verminderte Atemgeräusche. Obstruktive (Emphysem) und restriktive (Fibrose) Veränderungen in denselben Lungen führen zu erhaltenen Lungenvolumina, aber beeinträchtigtem Gasaustausch. Dementsprechend zeigen sich in Lungenfunktionstests normale Spirometrieergebnisse und Lungenvolumina, jedoch eine verminderte DLCO [23].

### Histopathologische Präsentation und radiologische Korrelation

**Abb. 5:**
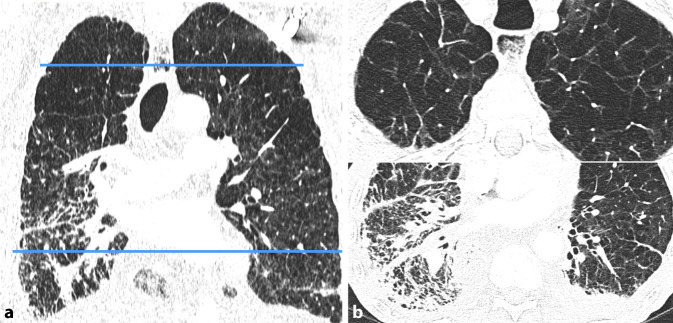
Kombinierte pulmonale Fibrose mit Emphysem (*CPFE*, **a**) mit entsprechend konfluierendem, apikal betontem zentrilobulärem Emphysem (**b** *oben*) sowie basal verdickten Septen und Bronchiektasien im Sinne von fibrotischen Veränderungen (**b** *unten*)

In den apikalen Lungenabschnitten überwiegen in der Regel emphysematöse Veränderungen, während sich die fibrotischen Strukturveränderungen auf die unteren Lungenbereiche konzentrieren. Abb. 5 veranschaulicht diese Verteilung. Es existiert keine eindeutige Definition, die für die Diagnosestellung maßgeblich ist. Dennoch haben Cottin et al. [9] versucht, eine solche Definition zu entwickeln. Hierbei wurden eine wissenschaftliche als auch eine klinische Definition formuliert. Die wissenschaftliche Definition von CPFE bezeichnet das Vorhandensein sowohl von Lungenfibrose als auch von Emphysem in den Lungen eines Patienten, welche in der HRCT klar ersichtlich sind. Emphysem wird durch gut abgegrenzte Bereiche mit geringer Dichte, begrenzt durch dünne Wände oder ohne Wand, definiert und betrifft mindestens 5 % des gesamten Lungenvolumens. Ebenso müssen fibrotische Veränderungen jeglichen Subtyps auf dem HRCT nachweisbar sein. Ein Mindestausmaß an Fibrose bzw. eine Definition des Fibrosemusters gibt es nicht. Zusätzliche Kriterien für die Klassifikation der klinischen CPFE umfassen eine Emphysemausdehnung von ≥ 15 % des gesamten Lungenvolumens, relativ erhaltene Lungenvolumina und vermindertem DLCO sowie das Vorhandensein von präkapillärer pulmonaler Hypertonie [9]. In einigen Bereichen können pigmentierte alveoläre Makrophagen vorhanden sein, die RB-ILD oder DIP ähneln. In einigen Fällen können Emphysem und Fibrose im gleichen Lungengebiet auftreten. Dieses CT-Muster kann mit anderen zystischen Lungenerkrankungen wie der Lymphangioleiomyomatose und der pulmonalen Langerhans-Zell-Histiozytose (PLCH) verwechselt werden.

### Therapie und Prognose

Obwohl ein internationales Gremium im Jahr 2022 die Terminologie und Definitionen von CPFE veröffentlichte, steht eine Einigung über genaue diagnostische Kriterien und optimale Behandlungsstrategien noch aus. Pulmonale Hypertonie ist in der CPFE häufig und ein entscheidender Faktor für die Prognose. Die mittlere Überlebenszeit wird mit 6,1 Jahren angegeben, besser als bei Patienten mit alleiniger IPF, aber schlechter als bei Emphysem ohne Fibrose [9].

## Idiopathische pulmonale Fibrose vs. raucherassoziierte interstitielle Fibrose

Seit vielen Jahren besteht der Verdacht auf eine Verbindung zwischen Zigarettenrauchen und Lungenfibrose [3, 16]. In Studien zu idiopathischer Lungenfibrose (IPF) variieren die Prävalenzen je nach verwendeter Falldefinition und dem Anteil aktueller oder ehemaliger Raucher zwischen 41 und 83 Prozent. Am häufigsten sind Männer über 60 Jahren betroffen, genetisch Risikofaktoren können aber ein jüngeres Erkrankungsalter im Rahmen der familiären IPF bedingen [26]. Die HRCT zeigt ein UIP-Muster („usual interstitial pneumonia“), welches sich aus Honigwabenmuster sowie Retikulationen mit ggf. zusätzlichen Traktionsbronchiektasien zusammensetzt sowie insbesondere basal und subpleural verteilt ist. Ground-glass-Opazitäten sprechen gegen das Vorliegen eines UIP-Musters. Bei einem klassischen UIP-Muster wird eine Biopsie nur bei untypischer Klinik empfohlen [20]. Das histologische Merkmal der IPF ist ihre Heterogenität. Oftmals sind in derselben offenen Lungenbiopsie Bereiche mit normalem Lungengewebe, aktiver Entzündung oder Alveolitis und Fibrose vorhanden. In Bereichen mit fibrotischen Veränderungen finden sich zellfreies Kollagen, eine Hyperplasie der glatten Muskulatur und ein Honigwabenmuster (zystisch vergrößerte Bronchien mit metaplastischem bronchialem Epithel ausgekleidet; [2, 28]). Ohne Therapie beträgt die durchschnittliche Überlebenszeit etwa 2,5 bis 3,5 Jahre, wobei antifibrotische Medikamente wie Pirfenidone oder Nintedanib das Fortschreiten der Krankheit verlangsamen können.

Die raucherassoziierte interstitielle Fibrose (SRIF) ist eine spezifische Form chronischer interstitieller Fibrose, die häufig bei Zigarettenrauchern auftritt [17]. Sie ist durch gleichmäßige Verdickung der Alveolarsepten durch Kollagenablagerung mit minimaler Entzündung gekennzeichnet. Die Verteilung ist meist subpleural betont und mit einem Emphysem assoziiert. Die hyalinisierte Qualität des Kollagens unterscheidet SRIF von anderen Formen der Fibrose. Ein UIP-Muster ist in der SRIF nicht vorhanden [11]. In einer kürzlich durchgeführten Studie wurden in Lobektomie-Proben von Rauchern SRIF bei 45 % der Fälle identifiziert [17]. Das Durchschnittsalter betrug 65 Jahre, die Hälfte waren aktuelle Raucher, die andere Hälfte ehemalige Raucher.

Da die IPF mit einer deutlich niedrigeren 5‑Jahres-Überlebensrate einhergeht (SRIF 85,7 % vs. IPF 40,7 %), ist die Unterscheidung zwischen SRIF und IPF von wichtiger prognostischer Bedeutung. Insbesondere das UIP-Muster, welches nur bei der IPF vorhanden ist, spielt zur Unterscheidung eine wichtige Rolle [7].

Eine Übersicht der wichtigsten klinischen sowie histopathologischen und radiologischen Charakteristika der einzelnen Pathologien ist in Tab. 1 aufgeführt.

**Tab. 1:** Häufigste raucherassoziierte Lungenerkrankungen mit entsprechenden Charakteristika

Erkrankung	RB	RB-ILD	DIP	PLCH	CPFE
*Erkrankungsalter*	> 90 % aller starken Raucher	30–40 Jahre	30–60 Jahre	20–40 Jahre	60–80 Jahre
*Radiologisches Muster*	Keine Veränderungen Zentrilobuläre Noduli	Zentrilobuläre Noduli Ground-glass-Opazitäten Retikulationen	Flächige Ground-glass-Opazitäten	Zentrilobuläre Noduli mit Kavernen im Verlauf	Apikales Emphysem Basale Fibrose
*Zusätzliche bildgebende Befunde*	Verdickte Bronchialwände	Verdickte Bronchialwände	Dünnwandige Zysten Retikulationen Fibrose bei fehlender Behandlung	Bizarre Zysten Fibrose	Mindestens 5 % Emphysem
*Verteilung*	Apikale Betonung	Apikale Betonung	Basale Betonung	Apikale Betonung	Apikales Emphysem/basale Fibrose
*Histopathologisches Korrelat*	Anschoppung von pigmentierten Makrophagen in den respiratorischen Bronchioli	Anschoppung von pigmentierten Makrophagen in den respiratorischen Bronchioli mit peribronchialer Inflammation	Exzessive Anschoppung von pigmentierten Makrophagen in Alveolen	Granulomatöse Entzündung, Langerhans-Zellen Zystische Dekonfiguration der Bronchialwände, sternförmige Narben/Fibrose	Emphysematöse sowie fibrotische Veränderungen derselben Lunge
*Pulmonale Funktionstests*	Normal	DLCO reduziert	DLCO reduziert	Obstruktion/Restriktion (gemischt)	Obstruktion/Restriktion (gemischt) DLCO/Volumina reduziert
Differenzialdiagnosen*	EAA Follikuläre Bronchiolitis	EAA DIP NSIP	RB-ILD EAA NSIP Atypische Infektionen	Sarkoidose Silikose Tuberkulose Metastasen LAM	IPF

*RB* respiratorische Bronchiolitis, *RB-ILD* respiratorische Bronchiolitis mit interstitieller Lungenerkrankung, *DIP* desquamative interstitielle Pneumonie, *PLCH* pulmonale Langerhans-Zell-Histiozytose, *CPFE* kombinierte pulmonale Fibrose mit Emphysem, *IPF* idiopathische pulmonale Fibrose, *DLCO* Kohlenmonoxid-Diffusionskapazität, *EAA* exogen-allergische Alveolitis, *NSIP* nichtspezifische interstitielle Pneumonie, *LAM* Lymphangioleiomyomatose

*In Bezug auf das radiologische Muster

## Fazit für die Praxis

- Die hochauflösenden Computertomographie (HRCT) spielt eine entscheidende Rolle bei der Identifikation charakteristischer Merkmale von rauchassoziierten interstitiellen Lungenerkrankungen (SR-ILD).
- Bei Überlappungen und Koexistenz mit anderen Lungenerkrankungen ist eine integrierte klinische, radiologische und pathologische Herangehensweise entscheidend für eine präzise Diagnose.
- Die verschiedenen Krankheitsbilder stellen häufig ein Spektrum dar, das von entzündlichen Prozessen über Destruktion bis hin zu Fibrose reicht.
- Die einheitliche Therapieempfehlung beinhaltet den Rauchstopp; weitere Therapien sind meist symptomatisch und verhindern nicht das Fortschreiten der Erkrankung.

## Abkürzungen

CPFE: Kombinierte pulmonale Fibrose mit Emphysem
DIP: Desquamative interstitielle Pneumonie
ILD: Interstitielle Lungenerkrankung
IPF: Idiopathische pulmonale Fibrose
PFT: Pulmonale Funktionstests
PLCH: Pulmonale Langerhans-Zell-Histiozytose
RB: Respiratorische Bronchiolitis
SR: Raucherassoziiert
